# Genetic Variance of Metabolomic Features and Their Relationship With Malting Quality Traits in Spring Barley

**DOI:** 10.3389/fpls.2020.575467

**Published:** 2020-10-19

**Authors:** Xiangyu Guo, Pernille Sarup, Jens Due Jensen, Jihad Orabi, Nanna Hellum Kristensen, Frans A. A. Mulder, Ahmed Jahoor, Just Jensen

**Affiliations:** ^1^Center for Quantitative Genetics and Genomics, Aarhus University, Tjele, Denmark; ^2^Nordic Seed A/S, Odder, Denmark; ^3^Department of Chemistry and Interdisciplinary Nanoscience Center (iNANO), Aarhus University, Aarhus, Denmark; ^4^Department of Plant Breeding, Swedish University of Agricultural Sciences, Alnarp, Sweden

**Keywords:** barley, malting quality, metabolomic features, heritability, correlation

## Abstract

Barley is the most common source for malt to be used in brewing beer and other alcoholic beverages. This involves converting the starch of barley into fermentable sugars a process that involves malting, that is germinating of the grains, and mashing, which is an enzymatic process. Numerous metabolic processes are involved in germination, where distinct and time-dependent alterations at the metabolite levels happen. In this study, 2,628 plots of 565 spring malting barley lines from Nordic Seed A/S were investigated. Phenotypic records were available for six malting quality (MQ) traits: filtering speed (FS), wort clearness (WCL), extract yield (EY), wort color (WCO), beta glucan (BG), and wort viscosity (WV). Each line had a set of dense genomic markers. In addition, 24,018 metabolomic features (MFs) were obtained for each sample from nuclear magnetic resonance (NMR) spectra for wort samples produced from each experimental plot. The genetic variation in the MFs was investigated using a univariate model, and the relationship between MFs and the MQ traits was studied using a bivariate model. Results showed that a total of 8,604 MFs had heritability estimates significantly larger than 0 and for all MQ traits, there were genetic correlations with up to 86.77% and phenotypic correlations with up to 90.07% of the significant heritable MFs. In conclusion, around one third of all MFs were significantly heritable, among which a considerable proportion had significant additive genetic and/or phenotypic correlations with the MQ traits (WCO, WV, and BG) in spring barley. The results from this study indicate that many of the MFs are heritable and MFs have great potential to be used in breeding barley for high MQ.

## Introduction

Although different types of starchy plants have been used for brewing, such as rice, wheat, maize, millet, and sorghum, barley is the most common source for malt to be used in brewing beer and other alcoholic beverages (Zhou, 2010). In the last decades, the amount of barley used for brewing has significantly increased (Zhou, 2010). Two-row spring barley with large grains is preferred for malting because of higher starch content and lower protein content compared to six-row winter barley (Hartmeier and Reiss, 2011). Malting quality (MQ) traits are important in spring barley, since they can be directly related to the quality of brewed beer and the amount of alcohol that can be made from the grain. MQ is composed of a series of traits, such as malt extract, malt protein, soluble protein, free amino nitrogen, diastatic power, apparent attenuation limit, wort viscosity (WV), alpha-amylase, beta-glucanase, wort beta-glucan, taste, flavor, haze, foam head retention, and antioxidants (Li et al., 2010). Because of complex inheritance and difficulty in phenotypic evaluation of these MQ traits, it is an expensive and labor-intensive process to measure MQ (Gao et al., 2004). For example, high extract yield (EY) can increase the amount of substrate available for fermentation, beta-glucan (BG) content should be low to avoid filtration problems caused by high WV (Bamforth, 2003, 2009). A detailed analysis of genetic variation in MQ traits in spring barley was provided by Sarup et al. (2020). In this previous study, a population of 1,329 spring barley lines from four breeding cycles were investigated and medium to high narrow sense heritabilities (0.31–0.65) were found for the MQ traits studied.

Brewing is a process to convert the starch of a cereal into alcohol and other fermentation products. The first stage of the brewing process is hydrolysis of the starch into fermentable sugars and the second stage is the conversion of these sugars into alcohol and carbon dioxide (Østergaard and Olsen, 2011). The first stage often involves malting, that is a germination of grains, and mashing, which is an enzymatic process (Hartmeier and Reiss, 2011). Numerous metabolic processes are involved in germination with final result in distinct and time-dependent alterations of the metabolite profiles (Frank et al., 2011).

Metabolites are typically intermediates of biochemical reactions, which connect complex interactions, cellular pathways, DNA, RNA, protein, and environmental stimuli (Jewett et al., 2006). Therefore, metabolomics, an approach that aims to identify and quantify the endogenous metabolites in a cell, provides a unique opportunity to functionally understand the physiological state of an organism (Gieger et al., 2008). All omics approaches, i.e., genomics, transcriptomics, proteomics, and metabolomics, are important tools which can be applied and utilized to investigate the biology of an organism (Roessner and Bowne, 2009). Compared with the codes of DNA, RNA, and proteins, the diversity in types and levels of metabolites is much greater, which means more complex analytical approaches are required to elucidate the elemental composition, the order of the atoms and the stereochemical orientation of individual metabolites (Fiehn, 2002). In order to analyze most metabolites simultaneously, a range of analytical technologies have been developed, including liquid chromatography-mass spectrometry (LC-MS), gas chromatography-mass spectrometry (GC-MS), nuclear magnetic resonance (NMR) spectroscopy, and enzyme assays (Lu et al., 2017). In metabolomic research, there are many advantages of NMR, such as high reproducibility, non-destructive, non-invasive, and quantitative nature of results, minimal need for sample preparation, and high efficiency, which allows high number of metabolites to be detected simultaneously in only a few minutes for each sample (Emwas, 2015). The signal intensities obtained from NMR could be treated as an indicator of biological sample metabolites and named as metabolomic features (MFs) (Aliakbari et al., 2019).

Different from animals, the growing ability of green plants primarily depends on their own photosynthetic and metabolic capacity because plants mostly produce their own organic compounds (Meyer et al., 2007). Therefore, metabolomics is of great importance in the plant field since plants collectively produce a very much larger array of metabolites than most other organisms such as animals and microorganisms (Saito and Matsuda, 2010). A comprehensive view of cellular metabolites can be provided by metabolomics. As metabolites participate in different cellular events, the physiological state of a cell can be represented by the metabolic profile obtained from metabolomics (Kumar et al., 2017). The rapid development of metabolomics has spurred its application in various fields, and contributes to the molecular and biological characterization of various organisms. For example, metabolomics can help to discover genes and pathways (Tohge et al., 2014; Hong et al., 2016) as well as understand the function of genes (Wen et al., 2015). Another utilization of metabolomics is to treat the metabolome as an objective proxy for phenotypic data, because metabolites directly link to phenotypes in biological systems (Daygon and Fitzgerald, 2013). When the MFs are treated as phenotypes, they can be influenced by genetic and environmental effects just as almost all other phenotypic traits. However, the number of studies where metabolomic profiles have been used as phenotypes and where the genetic variance in these phenotypes have been studied are limited (Wittenburg et al., 2013; Matros et al., 2017). A previous study explored the magnitude of the additive genetic effects on NMR MFs, and showed that the heritabilities of MFs were up to 0.52 and among MFs having heritabilities significantly different from 0, up to 12% have significant genetic correlation with growth traits in Holstein cattle (Aliakbari et al., 2019). Investigation of genetic variance in metabolomic data is expected to provide a better understanding of the extent by which variation in MFs is due to underlying genetic factors. Furthermore, with integrating metabolomic profile and genomic information, the genetic and physiological background of economical traits are also expected to be better understood.

Therefore, the objectives of this study were to (1) investigate genetic variation in MFs in malted spring barley; (2) study the genetic and phenotypic relationships between MFs and MQ traits.

## Materials and Methods

### Data

There were 565 spring barley malting lines from Nordic Seed A/S included in the current study. These lines were part of the standard breeding program of the commercial breeding company and were grown in two locations in Denmark and in each location harvested separately in 3 years (2014, 2015, and 2016). The fields were divided in trials consisting of smaller plots, and each trial was a randomized block-design with three replicates of each line (Nielsen et al., 2016). Therefore, the experimental design allowed testing conducted in a number of trials within each year-location subgroup. In 2014, 2015, and 2016, there were 140, 212, and 213 lines, respectively. In this breeding program, new crosses between selected elite parents are made every year. These lines go through several generations of selfing, and single seed descent lines are formed in fourth generation (F4). This is followed by an additional field growing year for multiplication in fifth generation (F5), then generate the sixth generation (F6) lines. The F6 lines are tested in standard field trials and one aspect of the many things recorded is MQ. In each year a new set of F6 lines enter the testing scheme. Thus, in general, lines are not repeated over years. In spring barley, parents used for crossing in commercial breeding programs are primarily elite lines from previous testing cycles and therefore tend to be genetically related. This can be seen easily from the fact that lines from all breeding cycles are in a single cluster in Supplementary Figure S1 where the first two principal components from a principal component analysis (PCA) on the genotypes describe 40% of the total genomic variance. Thus, lines in each consecutive breeding cycle (year) will tend to segregate in the same loci, and so that loci that are responsible for quantitative genetic variation in MQ and MFs tend to be the same across breeding cycles. Therefore, all samples and data belonged to the same program. Genotypic data from each line (Supplementary Table S1), records of six MQ traits from each plot (Supplementary Table S2), and MFs from each plot (Supplementary Table S3) are available in a publicly accessible repository (Guo, 2020). On average, there were 4.65 replicates available for each line, i.e., in total there were records from 2,628 plots available for analysis. Raw data collected from each plot were used instead of precorrection or summary statistics like line means. For each plot, a wort, which is the malt milled and extracted in water, was produced (Sarup et al., 2020) and used to measure MQ traits that included filtering speed (FS), wort clearness (WCL), EY, wort color (WCO), BG, and WV. FS was scored by measuring the height of the liquid surface in the glass 20 min after filtering had begun (cm flow-through in 20 min). WCL was evaluated visually at this step by scoring each filtrate from 1 to 3, where 1 was clear and 3 was opaque. EY was the percentage of dry matter. WCO was determined by spectrophotometer using the method of European Brewery Convention (EBC) (Bishop, 1966). After filtration, the wort samples were separated in two parts and all wort phenotypes were obtained according to the Analytica-EBC 2004 manual. Briefly, one sample of 25 ml of wort was used for WV (mPa⋅s, Analytical-EBC 8.4) and EY (Analytical-EBC 8.3). A second sample of 3–4 ml of wort was used for BG (mg/L, Analytical-EBC 8.13.1) and WCO (Analytical-EBC 8.5). Detailed description of MQ traits also can be found in a previously published study (Sarup et al., 2020).

Genotypic data were based on the Illumina iSelect9K barley chip and a total of 3,889 single-nucleotide polymorphism (SNP) markers were used after editing according to minor allele frequency more than 5% and missing markers less than 20%. These genotypic data were used to define additive genetic relationships between lines and used as a basis for the estimation of additive genetic variances and covariances. MFs were NMR data expressed as 24,018 intensities obtained from one-dimensional (1D) ^1^H NMR spectra, the intensities were integrated over small chemical shift (δ) intervals, expressed in parts per million (ppm) in the frequency range from 0.70 to 9.00 ppm.

In total, there were 2,628 samples originating from individual plots with records of six MQ traits and 24,018 MF intensities, the plots were from 565 lines, and each line had genomic information on 3,889 SNPs evenly spread over the genome.

### Extraction of Samples Used for NMR-Analysis

For each sample to be analyzed, exactly 0.050 g freeze dried malt was dissolved in 1 ml distilled water (H_2_O), which included 0.5 mg ⋅ ml^–1^ sodium trimethylsilyl propanesulfonate (DSS-d_6_; deuterated at all positions except for the methyl group). The DSS methyl signal will serve a dual role as NMR chemical shift reference (0.00 ppm) as well as internal concentration standard for NMR spectroscopy later. Samples were collected in 96-well containers, with one reference position that only contained DSS solution as a control. Each 96-well plate was then heated at 70°C for 60 min in a thermocycler with a shaking frequency of 800 rpm. After heating, samples were cooled in a water bath at room temperature for 60 min. To precipitate any remaining insoluble debris, plates were centrifuged for 20 min at 4,000 rpm. Subsequently, 700 μl of supernatant from each sample was then carefully pipetted to new 96-well plates, which were flash frozen, covered with parafilm, and transferred into a freezer drier under vacuum to sublimate all ice. The containers with dry frozen powder were then stored at −20°C.

Nuclear magnetic resonance samples were prepared by dissolving the dry material in each vile into 550 ml heavy water (D_2_O). Subsequently, each solution was transferred to a 5 mm NMR-tube and placed in 96-tube trays suitable for a Bruker SampleJet automatic sample changer. NMR metabolite spectra were then measured at 25°C on a Bruker 500 MHz spectrometer, using the zgpr pulse sequence (presaturation of residual HOD, followed by 90° excitation) with 16 scans, using an inter-scan delay of 2 s and 4.1 s acquisition. The NMR spectrum for each sample was recorded in under 2 min. Including sample changes and stabilization, ten samples were analyzed per hour.

### NMR Intensities

The spectra were processed using an in-house custom Matlab script (Haggart et al., 2019). Specifically, we applied an exponential apodization function equivalent to 0.5 Hz line-broadening, followed by Fourier transformation. All spectra were referenced to the DSS-d_6_ signal, automatically phased, and baseline corrected. We excluded the water peak (4.7–4.9 ppm) and the region of the added standard (−0.2 to 0.2 ppm). The raw data was normalized using the probabilistic quotient method (Dieterle et al., 2006), and the spectra were aligned using icoshift (Savorani et al., 2010; Vu and Laukens, 2013). The MFs were centered and standardized to a mean of 0 and standard deviation as 1 in order to refine variation that could be attributed to experimental sources and signal intensities (Aliakbari et al., 2019).

### Statistical Models and Methods

There were two steps involved in the statistical analyses. First, the genetic variation of MFs was investigated in univariate analyses (Model 1); second, the relationship between MFs and the MQ traits was studied by bivariate analyses (Model 2). These models were designed to decompose the total phenotypic variances into different components. In order to achieve this goal, phenotypic variances were allocated into components of genetic effects, environmental effects and their interactions. The genetic components included both genomic and non-genomic effects of the lines, the interaction components included both genomic and non-genomic by environmental effects, and the environmental component included batch effects and residual error.

In the first step, the following model was applied:

(Model 1) $$y_{m}=\mathrm{Xb}+Z_{g}\,g+Z_{l}\,l+Z_{i_{g}}\,i_{g}+Z_{i_{l}}\,i_{l}+Z_{t}\,t+e$$

where *y*_*m*_ referred to the vector of each MF, *b* was a vector of location × year × trial effects to correct for the differences might be caused by experimental location, year and trial, *g* was the vector of additive genomic effects for each line which could be explained by genomic markers, *l* was the vector of line effects for differences between lines not explained by additive genomic markers, *i*_*g*_ was additive genotype by environment (six location × year environments) interaction effects which accounted for the differences in genotype caused by various location × year environments, *i*_*l*_ was line by environmental interaction effects which accounted for the differences in line caused by various location × year environments, *t* was the vector of effects for batch of samples malted and mashed simultaneously which accounted for the environmental effects induced by the different batches, X, Z_*g*_, Z_*l*_, *Zi*_*g*_, *Zi*_*l*_, and Z_*t*_ were the corresponding design matrices allocating MF to *b*, *g*, *l*, *i*_*g*_, *i*_*l*_, and *t*, respectively, and *e* was a vector of residual terms which were the variances could not be explained by the other effects in the model. In this model, *b* was a fixed parameter, and *g*, *l*, *i*_*g*_, *i*_*l*_, *t*, and *e* were random parameters with $g∼N\,\left(0,G\,\sigma_{g}^{2}\right)$, $l∼N\,\left(0,I\,\sigma_{l}^{2}\right)$, $i_{g}∼N\,\left(0,d\,i\,a\,g\,\left(G,\cdots \,G\right)\,\sigma_{i_{g}}^{2}\right)$, $i_{l}∼N\,\left(0,I\,\sigma_{i_{l}}^{2}\right)$, $t∼N\,\left(0,I\,\sigma_{t}^{2}\right)$, $e∼N\,\left(0,I\,\sigma_{e}^{2}\right)$, and the random effects were assumed to be independent of each other. G denoted the genomic additive relationship matrix computed using genotypic data through VanRaden method 1 (VanRaden, 2008), ***diag*(*G*,⋯*G*)** denoted the diagonal matrix with G as block-diagonal elements in six location × year environments, and I denoted the identity matrix. By applying a G matrix, the effects of all markers were summarized as the genomic variance components and are equivalent of regressing each MF on all markers simultaneously (VanRaden, 2008). The genomic relationship matrix included in the model took advantages of the fact that varieties were related across years and locations. The extra variability from location × year to location × year was taken into account in the model via genotype by environment interactions and line by environment interactions. The genomic relationships were also used to ensure that all varieties obtained an estimate of the specific location × year effects via information from relatives.

In the second step, a bivariate model (Model 2) was used with Model 1 as sub-model for each trait (MF and MQ traits) involved in the analyses, and in which all dispersion parameters were expanded to 2 × 2 covariance matrices.

### Estimation of Variance Components and Population Parameters

The (co)variance components in all models described in the previous paragraph were estimated by restricted maximum likelihood (REML) using the DMU software package (Madsen and Jensen, 2013).

In the first step, the phenotypic variances of MFs were calculated as the sum of variance components (VCs) in Model 1:

$$\sigma_{P_{m}}^{2}=\bar{G}\,\sigma_{g}^{2}+\sigma_{l}^{2}+\bar{G}\,\sigma_{i_{g}}^{2}+\sigma_{i_{l}}^{2}+\sigma_{t}^{2}+\sigma_{e}^{2}$$

where $\bar{G}$ was the average diagonal of G. A genomic heritability, which is the part of additive genetic variance that can be explained by the markers, was calculated as estimated narrow sense heritability ($\hat{h^{2}}$) where $\hat{h^{2}}=\frac{\bar{G}\,\hat{\sigma_{g}^{2}}}{\hat{\sigma_{P_{m}}^{2}}}$. This is the heritability of a single plot measurement, and not a heritability of line means as occasionally done in plant breeding. In addition to the analyses of MFs, a simulation procedure was used in order to determine the null distribution of $\hat{h^{2}}$ in the specific statistical design used for MQ and MF records. There were 50,000 replicates in the simulation procedure. In each replicate, random numbers following normal distribution with zero mean and unit standard deviation were generated for all samples, i.e., only the residuals were simulated. The simulation procedure was carried out in order to estimate the null distribution of $\hat{h^{2}}$ using the exact same experimental design and the null hypotheses were that all VCs in the model except the residual were null. Then the $\hat{h^{2}}$ at 1% percentile of the simulated results was chosen as the significant cut-off point to determine the MFs with $\hat{h^{2}}$ significantly larger than 0.

In the second step, genetic and phenotypic correlations between each MQ traits and MFs were estimated for all cases with significant $\hat{h^{2}}$ for the MFs (MFh). In order to test whether the estimates of correlation were significantly different from 0 at 1% significance level on both sides of the normal distribution (*z* > 2.326), *z*-scores were calculated as follows (Altman, 1968):

$$z=\frac{x}{\sigma }$$

where *x* was the estimate of correlation and σ was the associated standard error.

## Results

In this study, genetic variation in MFs was estimated, and the genetic and phenotypic relationships between MFs and MQ traits were investigated.

### Descriptive Statistics for Malting Quality Traits

Table 1 gives the descriptive statistics of all the MQ traits analyzed in this study. It can be seen that there were 2,483 to 2,624 records analyzed for the six traits of MQ. The average for traits was 4.82 for FS, 1.12 for WCL, 82.50 for EY, 5.88 for WCO, 224.75 for BG, and 1.47 for WV. The coefficients of phenotypic variation (CVs) ranged from 4.09% for EY to 59.47% for BG. Among all the MQ traits, EY and WV had low CV which was below 5%, FS and WCO had moderate CV which was around 14%. The CV of WCL was relatively larger which was around 35% and the largest CV of around 60% was found in BG.

**TABLE 1:** Descriptive statistics for all malting quality traits.

Trait	No. of records	Unit	Average	S.D.	Min	Max	CV
FS	2,622	cm/20 min	4.82	0.66	1.40	6.30	13.66%
WCL	2,624	–	1.12	0.40	1.00	3.00	35.94%
EY	2,562	%	82.50	3.37	0.23	92.39	4.09%
WCO	2,619	EBC units	5.88	0.86	3.59	10.08	14.59%
BG	2,612	mg/L	224.75	133.65	70.00	1318.26	59.47%
WV	2,483	mPa⋅s	1.47	0.07	1.26	2.28	4.89%

*FS, filtering speed; WCL, wort clearness; EY, extract yield; WCO, wort color; BG, beta glucan; WV, wort viscosity; CV, coefficient of phenotypic variance.*

**FIGURE 1:**
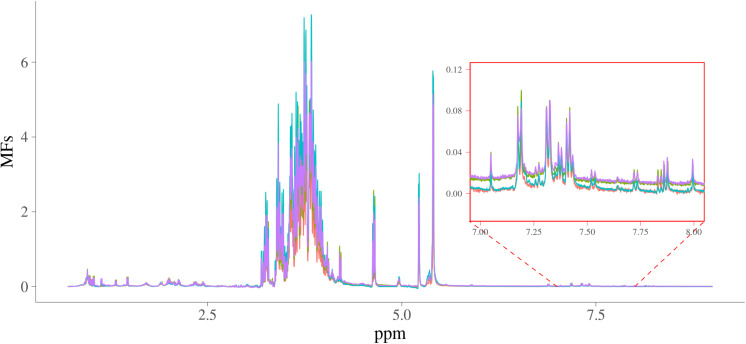
Spectra of four random samples. *x*-axis is NMR chemical shift (in ppm); *y*-axis are the intensities of the NMR signals; four samples are in different colors.

The MFs were for 2,628 samples each containing 24,018 intensities from 0.70011622 to 8.9999082 ppm in steps of around 0.0003364, and the intensities ranged from −0.266 to 17.1. The spectra of four random samples are plotted in Figure 1. In this figure, the intensities of spectra from 7 to 8 ppm were also shown as a zoomed-in plot.

### Estimation of Genetic Variation for Metabolomic Features

**FIGURE 2:**
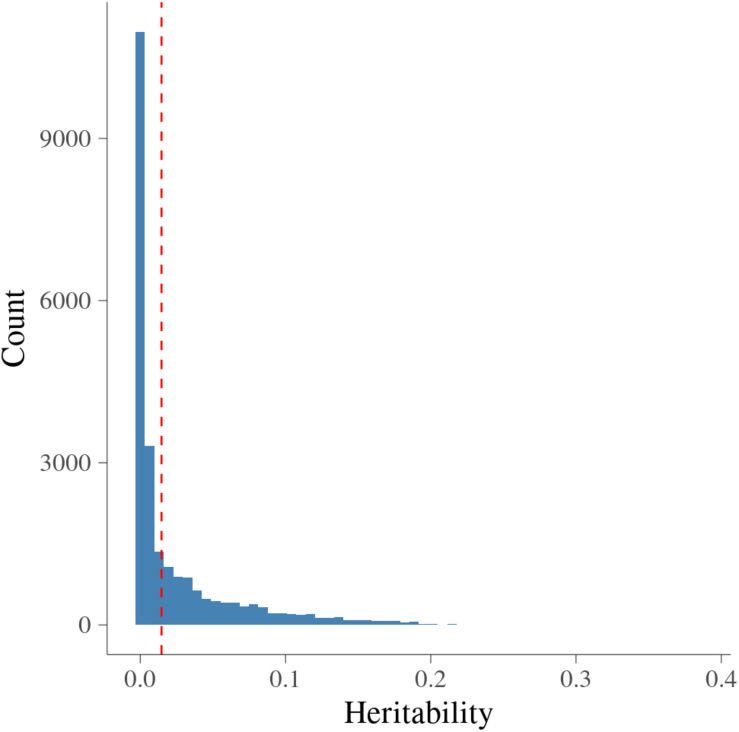
Histogram of heritability for metabolomic features. *x*-axis is heritability from metabolomic features; vertical dashed red line is the significance level at 1% at each side.

Figure 2 presents the estimated heritability of MFs. The estimates of heritability for 24,018 MFs ranged from 0 to 0.38 with average (±S.D.) as 0.025 (±0.041). There were around 0.3% MFs having **$\hat{h^{2}}$** larger than 0.2, showing medium heritability. The null distribution of heritability based on simulations with false discovery rate (FDR) of 0.05 were in the interval [0, 0.034]. The cut-off point corresponding to a significance level of 1% was 0.015. Therefore, there were 8,604 MFs (35.82%) having **$\hat{h^{2}}$** larger than 0.015, and were treated as MFs with significant additive genetic variance.

**FIGURE 3:**
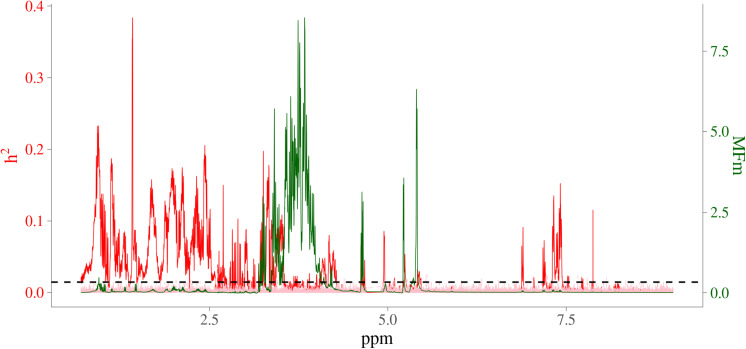
Heritability and average NMR intensities for metabolomic features. Red line is heritability from real metabolomic features; pink line is the null distribution of heritability from simulated data all not residual variance assumed 0; green line is average NMR intensities (before centering and standardizing); horizontal dashed black line is the significant level at 1%; *x*-axis is NMR chemical shift (in ppm); red *y*-axis is heritability; green *y*-axis are the intensities of the NMR signals, which are proportional to the amounts of the metabolites that give rise to the signals.

**FIGURE 4:**
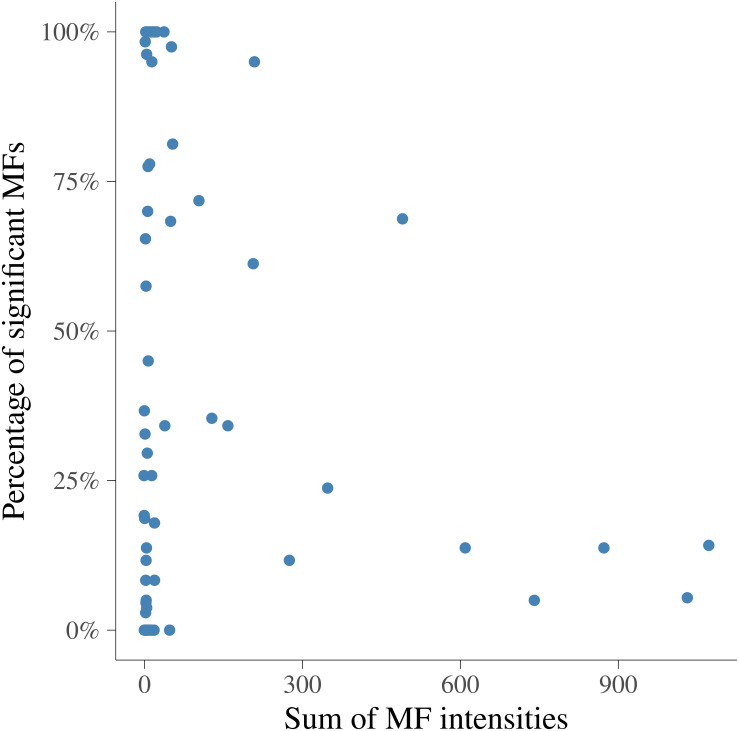
Relationship between the sum of metabolomic feature intensities and the proportion of significant heritable metabolomic features in 100 intervals. *x*-axis is the sum of metabolomic feature intensities in each interval, *y*-axis is the percentage of metabolomic feature having heritabilities significantly different from 0.

In order to visualize the MF intensities and their heritabilities, the heritability estimates for each MF and unstandardized mean intensities of MFs were plotted. Figure 3 presents the pattern of average MF intensities across all the samples (MFm) together with their **$\hat{h^{2}}$**. In Figure 3, the pattern of MFs was not necessarily consistent with the pattern of their **$\hat{h^{2}}$**. For example, the maximum **$\hat{h^{2}}$** (0.38) was at 1.423446 ppm, while the maximum MFm (8.55) was at 3.834322 ppm. To investigate the relation between the intensity and heritability, the entire range of NMR profiles was equally divided into 100 intervals and the proportion of MFs having **$\hat{h^{2}}$** significantly different from 0 among all the MFs in each ppm interval were plotted versus the sum of MF intensities in corresponding interval as shown in Figure 4. Each point in this figure shows in each interval of ppm, the relationship between the total MF intensities and the percentage of significant heritable MFs. It can be observed that there was no clear linear relationship between the proportion of significant heritable MFs and the MF intensities.

### Relationship Between Metabolomic Features and Malting Traits

In the second step, bivariate analyses were carried out to investigate the relationship of MFs having significant heritability (MFh) with each of the MQ traits. The statistical significance of correlation indicates the relationship between MQ traits and MFs rather than the sign of the correlation.

**FIGURE 5:**
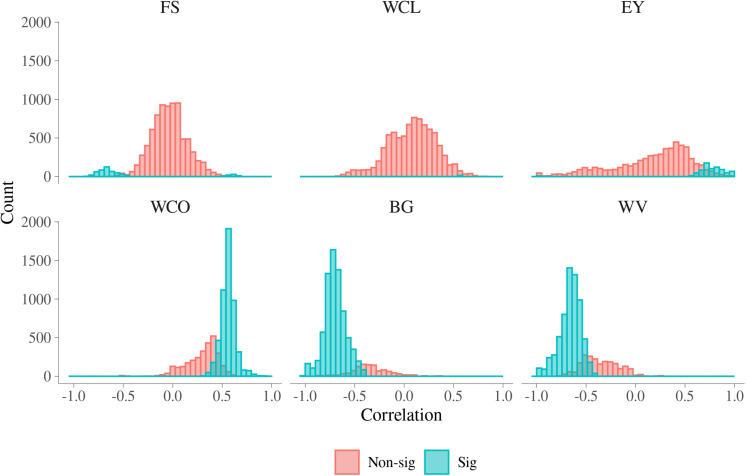
Additive genetic correlations for malting traits with significant metabolomic features. FS, filtering speed; WCL, wort clearness; EY, extract yield; WCO, wort color; BG, beta glucan; WV, wort viscosity.

In Figure 5, histograms of the estimated additive genetic correlations between MFh and six MQ traits are presented. In Supplementary Table S4, a summary for the range of additive genetic correlations and the percentage of significant correlations between the MFh and the six MQ traits is presented. For BG, the additive genetic correlation with MFh ranged from **−**1.000 to 0.349 and 86.77% of these correlations were significantly different from 0. The additive genetic correlations between BG and MFh tended to be significant for correlations lower than **−**0.4. The proportion of significant additive genetic correlations between WV with MFh were 75.05%, which was the second largest among all six MQ traits. Similar with BG, the additive genetic correlations between WV and MFh tend to be significant for correlations lower than **−**0.4. For WCO, 64.53% of the additive genetic correlations with MFh tended to be significant for correlations higher than 0.3. Whereas for three other traits, i.e., EY, FS, and WCL, the proportion of significant genetic correlation with MFh were much smaller (less than 12%) compared with the three traits mentioned above. For example, for WCL, the additive genetic correlation with MFh ranged from **−**0.694 to 0.842 and only 0.19% of these correlations were significantly different from 0.

**FIGURE 6:**
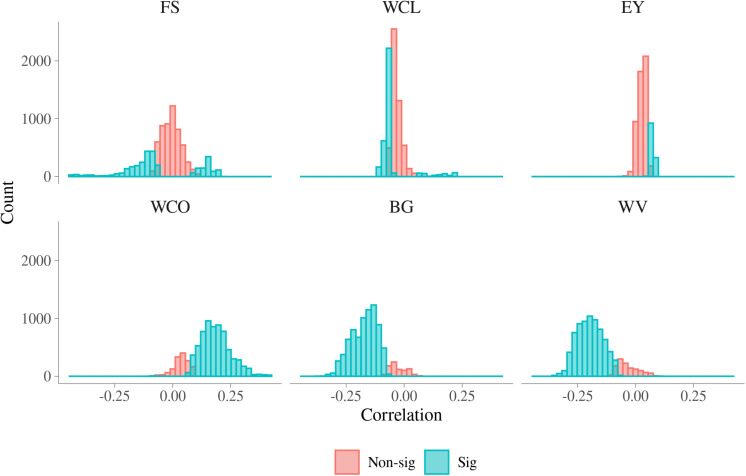
Phenotypic correlations for malting traits with significant metabolomic features. FS, filtering speed; WCL, wort clearness; EY, extract yield; WCO, wort color; BG, beta glucan; WV, wort viscosity.

Histograms of the estimated phenotypic correlations between MFh and six MQ traits are presented in Figure 6. A summary of the range of phenotypic correlations and the percentage of significant correlations between MFh and the six MQ traits is presented in Supplementary Table S5. The magnitude of phenotypic correlation was smaller than genetic correlation for each trait, while the number of phenotypic correlations significantly different from 0 was larger than the number of significant genetic correlations. This is mainly because phenotypic correlations are more accurately estimated than genetic correlations due to estimates of phenotypic correlations had lower standard error than estimates of genetic correlations. As with genetic correlation, a high proportion of the phenotypic correlations were significantly different from 0 for BG, WV, and WCO (90.07%, 85.93%, and 82.65%, respectively). The phenotypic correlations between BG and MFh ranged from **−**0.364 to 0.067, which was much narrower than the corresponding additive genetic correlations, and the significant phenotypic correlations tend to be significant for correlations with absolute value larger than 0.1. Similar to BG, the phenotypic correlations between WV and MFh ranged from **−**0.358 to 0.099, which was also much narrower than the corresponding additive genetic correlations, and the phenotypic correlations also tended to be significant for correlations lower than **−**0.1. The phenotypic correlations between WCO and MFh, which ranged from **−**0.100 to 0.416, tend to be significant for correlations higher than 0.1. Different from additive genetic correlations, the lowest significant phenotypic correlations were found for EY (19.72%), followed by FS (35.59%), and WCL (39.76%).

## Discussion

The current study analyzed the genetic variance in 24,018 individual MFs using univariate models, and then investigated genetic and phenotypic correlations between significantly heritable MFs and MQ traits in spring barley. A previous study of Sarup et al. (2020) reported the narrow sense heritability of line means for the MQ traits investigated in the current study. Specifically, the narrow-sense heritabilities of line means were 0.31 for FS, 0.56 for WCL, 0.51 for EY, 0.64 for WCO, 0.55 for BG, and 0.49 for WV (Sarup et al., 2020).

### Heritability of Metabolomic Features

In this study, the estimates of heritability for all MFs were low to medium. There were 35.82% MFs that were significantly heritable. The heritability of MFs measured on blood extracted from 843 male Holstein calves were investigated by Aliakbari et al. (2019), where 30,914 MFs were analyzed. There, the estimated heritabilities ranged from 0 to 0.52, which was wider than in the current study, but there were only 3.36% (1,040 MFs) significantly different from 0. Among all the MFs in this study, 3.57% had moderate heritability from 0.2 to 0.5 and 0.04% had high heritability larger than 0.5. Although the proportion of MFs having heritability larger than 0.2 in the current study was smaller than in the previous study on cattle, the proportion of significant heritability estimates was larger due to the large and more powerful experimental design used in this study. The effect of this was that the standard errors of estimates were much smaller in our study. The number of MFs with no significant heritability may also be influenced by including regions of the spectra with no biological signal. In future studies, it could also be considered to identify these areas and exclude them from the analysis in order to reduce noise in the metabolomic data.

From the comparison between patterns in estimates of heritability and the pattern in MFs intensity, no clear relationship was observed between magnitude of intensities and their magnitude of heritabilities. For example, for the MFs between 2.75 ppm to 2.85 ppm, there were multiple high peaks for the MF intensities, while the estimates of heritability in this interval were not high (although there were some small peaks just above the chosen level of significance). For the MFs between 0.50 ppm to 1.00 ppm, the intensities were low but there were several MFs having medium heritability and even the highest estimated heritability was located in this area. Although an identification of the molecules responsible for the MFs was not attempted, this region typically includes methyl groups from lipid ends and beta-branched amino acids. The medium heritabilities for these MFs indicated that this area represented biological compounds that were under considerable influence of additive genetic effects. The areas with highly heritable MFs represent typical amino acids such as tyrosine, phenylalanine and alanine. This pattern was also observed in the previous study especially for the range from 1 to 6 ppm, where most of the significant heritabilities appeared (Aliakbari et al., 2019).

Among all the 24,018 MFs analyzed in the current study, the heritabilities of 64.18% MFs were not significantly different from 0. It is not surprising that such a large proportion of MFs were not significantly heritable, because the entire NMR spectra were analyzed, which means that the areas with low or even no signal from metabolites were included and analyzed. The wide range of frequency (0.70 ppm to 9.00 ppm) of the NMR profile examined in this study could also be a factor. In the setting of the NMR spectral window, all relevant signals were expected to be detected. Some regions do not show (many) signals, for example in the right side of the spectra in Figure 3. This is typical for NMR metabolite spectra, with numerous aliphatic compounds contributing to the region 0–5 ppm, and far fewer aromatic compounds appearing around 6–7 ppm. The region from 3 to 4.5 ppm in the NMR spectrum is dominated by maltose, but also contains other sugars (e.g., glucose), while they did not show powerful heritabilities. It is, therefore, reasonable to include only those MFs with significant heritabilities for further analysis together with the MQ traits.

The definition and the estimation of heritability in plant breeding are complicated and diverse across studies since the observational units varies from individual plants to means of a genotype tested across different environments (Holland et al., 2003). Heritability estimated on the basis of plot and line mean are two often reported estimates, and depend on the units for measurement of phenotypic variances. Estimates of heritability on the plot basis are always smaller than the ones on the basis of line mean, since the estimates of phenotypic variance on the basis of plot are larger than those on the basis of line mean. This is because when computing estimated phenotypic variance, the variances due to interaction and error are divided by the corresponding numbers of observations (You et al., 2016), which in case of plot heritability is one. The metabolomic profile investigated in this study was for the wort sample on the basis of each plot, therefore, the estimates of heritability studied in our study was based on the plot level instead of line means.

In addition to plant species and livestock, there have been several studies on heritability in human metabolites (Nicholson et al., 2011; Kastenmüller et al., 2015; Frahnow et al., 2017). A very recent study reviewed relevant genetic analysis for metabolites studies published between November 2008 and October 2018, and highlighted the importance of common genetic variants for metabolite levels (Hagenbeek et al., 2020). The authors argued that, on average, around half of total phenotypic differences in metabolite levels was caused by genetic variance (with a range from 0 to 0.8), though heritability estimates differed across metabolite classes (Hagenbeek et al., 2020). The reason for the larger average heritability obtained in the human studies compared with the current study could be due to the fact that we studied the whole MFs profile so that some noise was included, while the human studies investigated quantified and identified metabolites.

There were two locations and 3 years involved in this study, and MFs for same variety can vary across different environments (location × year). Therefore, the model used in current study included also additive genotype by environment interaction effects (*i*_*g*_) and line by environment interaction effects (*i*_*l*_). The magnitudes of *i*_*g*_ and *i*_*l*_ are plotted as relative variance components (RVCs) in Supplementary Figure S2. RVCs of *i*_*g*_ ranged from 0 to 0.18 and RVCs of *i*_*l*_ range from 0 to 0.35, which indicate that environment did have differential effects on MFs. Therefore, in our study, the model which considering location × year environmental effects, did decompose the total phenotypic variance of MFs properly and extract variance of additive genomic effects (equivalent to *h*^2^) as well as genotype by environmental effects.

### Correlation Between Metabolomic Features and Malting Traits

There were 8,604 MFs having a heritability estimate significantly different from 0 and they were selected for a bivariate analysis together with each of the MQ traits. Each of the significant MFs was analyzed together with each trait, so that a total of 51,624 (8,604 × 6) bivariate analysis were carried out. Both genetic and phenotypic correlations were investigated in each of the bivariate analysis.

For all the traits, the average magnitude of phenotypic correlation between traits and MFs was smaller than the genetic correlation, while the proportion phenotypic correlations that were significant was larger than the proportion the genetic correlations that were significant. In the study on Holstein calves (Aliakbari et al., 2019) the magnitude of phenotypic correlations between traits and MFs was smaller than the corresponding genetic correlations, similar to what was observed in the current study. For example, the phenotypic correlation between body weight and MFs ranged from −0.16 to 0.20, while the genetic correlations ranged from −0.77 to 0.57 (Aliakbari et al., 2019). However, the difference in proportion of significant phenotypic and genetic correlations were reversed. In all cases investigated in the previous study, the proportion of phenotypic correlations that were significant was smaller compared with the corresponding additive genetic correlations (Aliakbari et al., 2019). The less significant genetic correlations between MFs and MQ traits could be related to the smaller heritability of MFs in the current study.

Among six MQ traits, WCO, WV, and BG displayed a high proportion of correlation (both genetic and phenotypic) with MFs, whereas EY, FS, and WCL exhibited a lower fraction. This indicates the larger impact of genetic effects on the relation between the metabolome and the traits of WCO, WV, and BG compared to EY, FS, and WCL. This might be useful to develop more efficient selection indices when formulating breeding plans for WCO, WV, and BG. The relationship between metabolites and MQ traits in barley was also investigated in a previous study, where non-targeted LC-MS metabolite profiling was applied (Heuberger et al., 2014). This previous study showed that barley and malt metabolites correlated with multiple MQ traits and both metabolites and quality traits covaried based on genetic and environmental parameters (Heuberger et al., 2014). The acquisition of quality phenotypes can be expensive, and when this is the case, high throughput phenotypes, such as information from NMR metabolomics, might help to decrease the expense or increase the speed of phenotyping (Hayes et al., 2017). A previous study showed that incorporation of NMR phenotypes in a multi-trait approach increased the accuracy of genomic prediction for quality traits in wheat (Hayes et al., 2017). Given the significant correlation between MFs and several MQ traits, incorporating NMR information could be expected to facilitate more efficient selection for MQ in spring barley.

The technology of NMR spectroscopy was used in this study to profile the metabolome in wort samples. Compared to the mass spectrometry (MS)-based metabolomic methods, LC-MS and GC-MS, NMR spectroscopy is very efficient for studies with large sample sizes. Though the price of the NMR instrument is not lower when compared to GC-MS and LC-MS, the NMR approach (i) needs virtually no preparation or derivatization, (ii) the process for measurement is faster and non-destructive, (iii) the procedure is highly reproducible, (iv) the output data is quantitative, (v) the output is comparable to data from other instruments, as all metabolites present in each sample are captured at the same time, (vi) there is no drift in the instrument. Collectively, these attributes strongly advocate the applicability of NMR metabolomics for further studies (Holzgrabe et al., 2005; Emwas, 2015). The main drawback of NMR compared to the MS based method is the poor sensitivity. However, the statistical correlations obtained in the current study demonstrated that for the purpose of estimating barley MQ, this is not a consequential shortcoming. Therefore, because of the many advantages of applying NMR in metabolomic analysis of large sample sizes, this approach is suggested when there are large amount of samples to be analyzed either in practical industrial and breeding applications or in theoretical research.

The models used in the current study implied estimation of effects from each marker as such models were equivalent of regressing each MF on all markers simultaneously (VanRaden, 2008; Legarra et al., 2018). Therefore, a significant genetic correlation between MFs and a MQ trait in the same population were a comprehensive demonstration of a genetic link between the two. In addition, in this study we utilized around 24K individual MFs. There was no one to one relationship between the MFs and specific metabolites. Each MF might contain the signal of more than one metabolite and most metabolites would contribute to the signal in more than one MF. In general MQ traits are highly polygenic (Sarup et al., 2020), therefore, summarizing the effects of all markers as the genomic variance components is a good way to investigate the genetic relationship between MFs and MQ.

## Conclusion

In this study, records of six MQ traits and MFs for 565 spring malting barley lines grown in two locations and harvested separately in 3 years were studied. On average, each line was replicated 4.65 times. Additive genetic relationships between lines were estimated from a set of dense markers covering the whole genome.

Genetic variation in MFs were investigated using a univariate model for each MF. The estimates of heritability for all the MFs ranged from 0 to 0.38. Among 24,018 MFs, 8,604 were significantly heritable and were selected for further bivariate analysis to estimate their genetic and phenotypic correlation with MQ traits. The large proportion of MFs having heritabilities not significantly different from 0 likely resulted from the inclusion of areas the investigated NMR spectra that had no biological signals. WCO, WV, and BG exhibited a high proportion of significant genetic and phenotypic correlation with selected MFs, while EY, FS, and WCL showed a low proportion. This indicates the large impact of genetic effects on the relation between the metabolome and WCO, WV, and BG, and might be useful to develop more efficient selection indices when formulating breeding plans for these traits. The profile of the metabolome in wort samples were produced by NMR, which has many advantages, e.g., low labor intensity, high reproducibility, fast, and non-destructive. With these advantages, this approach is suitable for large metabolomic data profiling both in practical industrial applications and in theoretical researches.

In conclusion, around 36% of MFs were significantly heritable, among which many were correlated with MQ traits of WCO, WV, and BG in spring barley. The results from this study indicate that many of the MFs are heritable and MFs have great potential to be used for more efficient selection for MQ in spring barley.

## Data Availability Statement

The datasets presented in this study can be found in online repositories. The names of the repository/repositories and accession number(s) can be found in the article/Supplementary Material.

## Author Contributions

NK carried out malting and extraction for NMR analysis. NK, PS, and FM developed the protocol for metabolite NMR analysis. NK, JDJ, and JO provided the genotypic and phenotypic data. XG implemented and carried out the statistical analysis, interpreted the results, and drafted the manuscript. NK, JDJ, AJ, JJ, and FM contributed to the experimental design. PS, JJ, and XG developed the statistical models. All authors participated in interpreting the results and read and approved the final manuscript.

## Conflict of Interest

PS, JDJ, JO, and AJ are employed by the Nordic Seed A/S, and NK is employed by SEGES. The funders had no influence on the study design or choice of analysis methods. The remaining authors declare that the research was conducted in the absence of any commercial or financial relationships that could be construed as a potential conflict of interest.

## Supplementary Material

Supplementary Figure 1 First two principal components from a principal component analysis on the genotype.

Supplementary Figure 2 Estimated relative variance components in metabolomic features. *g* is additive genomic effects; *i*_*g*_ is additive genotype by environment interaction effects; *i*_*l*_ is line by environment interaction effects; *x*-axis is NMR chemical shift (in ppm); *y*-axis is relative variance components (RVCs).

Supplementary Table 1 Genotypic data for each line.

Supplementary Table 2 Phenotypic data for malting quality traits.

Supplementary Table 3 Metabolomic data for each plot.

Supplementary Table S4 Descriptive statistics for genetic correlations between 8,604 significantly heritable metabolomic features and malting quality traits.

Supplementary Table S5 Descriptive statistics for phenotypic correlations between 8,604 significantly heritable metabolomic features and malting quality traits.

## Abbreviations

BG: beta glucan
CV: coefficient of phenotypic variance
EBC: European Brewery Convention
EY: extract yield
F4: fourth generation
F5: fifth generation
F6: sixth generation
FDR: false discovery rate
FS: filtering speed
GC-MS: gas chromatography-mass spectrometry
LC-MS: liquid chromatography-mass spectrometry
MFh: metabolomic features having significant heritability
MFm: average metabolomic features intensity across all the samples
MFs: metabolomic features
MQ: malting quality
MS: mass spectrometry
NMR: nuclear magnetic resonance
PCA: principal component analysis
ppm: parts per million
REML: restricted maximum likelihood method
RVC: relative variance component
SNP: single-nucleotide polymorphism
VCs: variance components
WCL: wort clearness
WCO: wort color
WV: wort viscosity.
