# Supplementary figures and images for: Genetic Variance of Metabolomic Features and Their Relationship With Malting Quality Traits in Spring Barley
Source: Front Plant Sci. 2020 Oct 19;11:575467. doi: 10.3389/fpls.2020.575467 (PMC7604292; doi:10.3389/fpls.2020.575467)

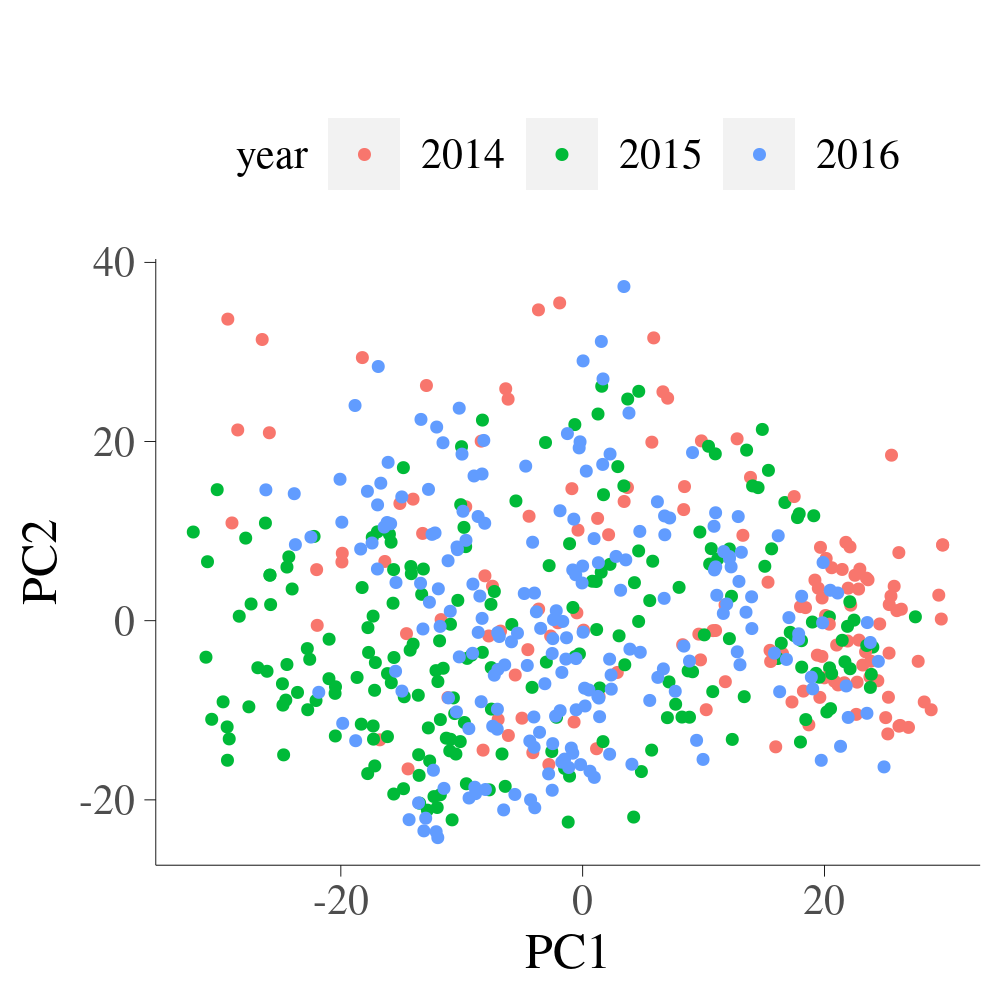

Supplement: Supplementary Figure 1 — First two principal components from a principal component analysis on the genotype. [file Image_1.JPEG]

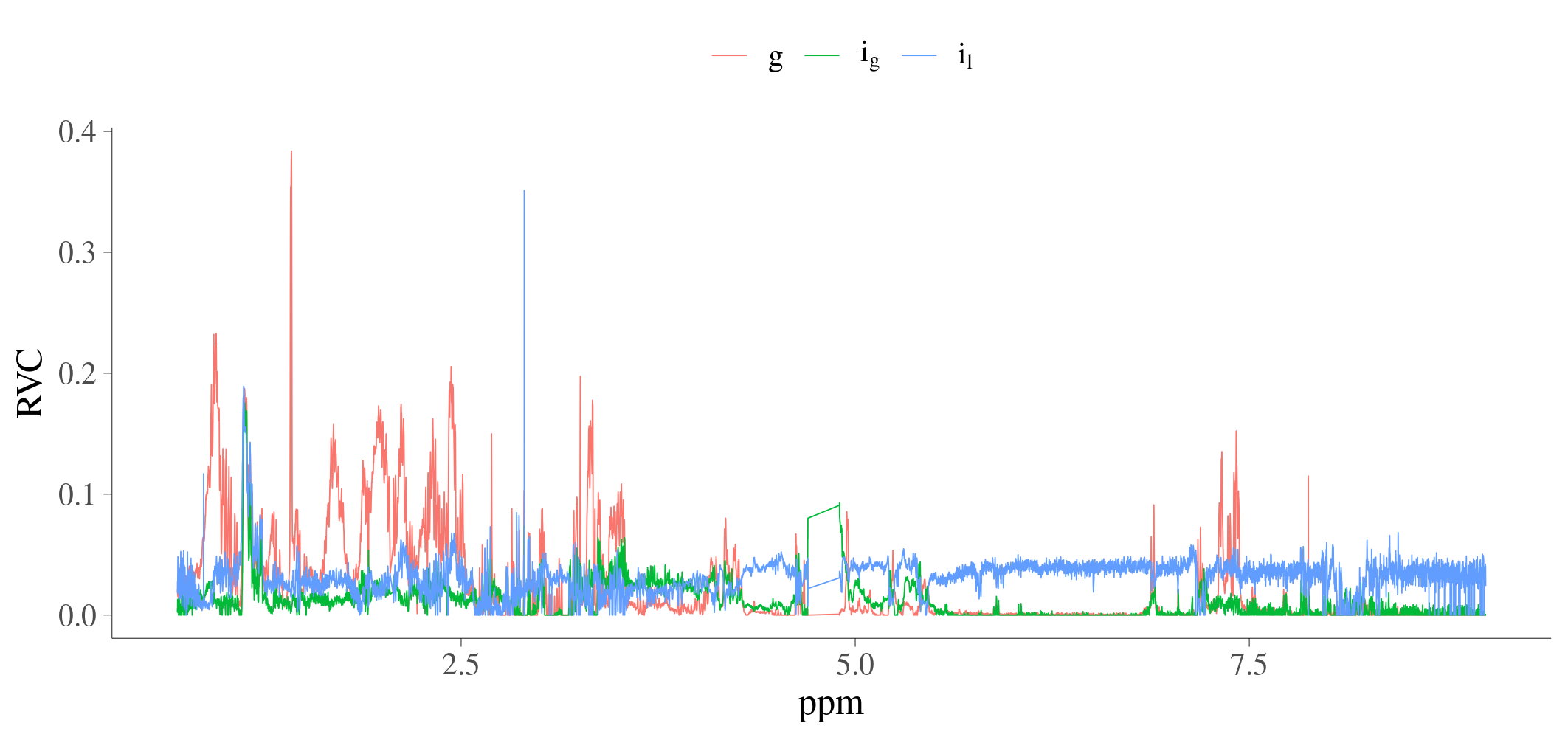

Supplement: Supplementary Figure 2 — Estimated relative variance components in metabolomic features. g is additive genomic effects; ig is additive genotype by environment interaction effects; il is line by environment interaction effects; x-axis is NMR chemical shift (in ppm); y-axis is relative variance components (RVCs). [file Image_2.JPEG]
