# Supplementary material for: Genetic Variance of Metabolomic Features and Their Relationship With Malting Quality Traits in Spring Barley
Source: Front Plant Sci. 2020 Oct 19;11:575467. doi: 10.3389/fpls.2020.575467 (PMC7604292; doi:10.3389/fpls.2020.575467)
Supplement: Supplementary Table S4 — Descriptive statistics for genetic correlations between 8,604 significantly heritable metabolomic features and malting quality traits. [file Data_Sheet_1.docx]

Supplementary Material

# Supplementary Data

**Table S1** Genotypic data for each line

**Table S2** Phenotypic data for malting quality traits

**Table S3** Metabolomic data for each plot

# Supplementary Tables

**Table S4** Descriptive statistics for genetic correlations between 8,604 significantly heritable metabolomic features and malting quality traits

| Trait | Average | S.D. | Min | Max | Significant^*^ |
| --- | --- | --- | --- | --- | --- |
| FS | -0.063 | 0.229 | -0.860 | 0.659 | 6.09% |
| WCL | 0.081 | 0.241 | -0.694 | 0.842 | 0.19% |
| EY | 0.247 | 0.418 | -1.000 | 1.000 | 11.88% |
| WCO | 0.468 | 0.168 | -0.526 | 0.960 | 64.53% |
| BG | -0.650 | 0.172 | -1.000 | 0.349 | 86.77% |
| WV | -0.585 | 0.183 | -1.000 | 0.255 | 75.05% |

FS is filtering speed, WCL is wort clearness, EY is extract yield, WCO is wort color, BG is beta glucan, WV is wort viscosity; ^*^ Correlation that significantly different from 0.

**Table S5** Descriptive statistics for phenotypic correlations between 8,604 significantly heritable metabolomic features and malting quality traits

| Trait | Average | S.D. | Min | Max | Significant^*^ |
| --- | --- | --- | --- | --- | --- |
| FS | -0.025 | 0.100 | -0.437 | 0.209 | 35.59% |
| WCL | -0.041 | 0.045 | -0.112 | 0.222 | 39.76% |
| EY | 0.036 | 0.024 | -0.085 | 0.095 | 19.72% |
| WCO | 0.161 | 0.082 | -0.100 | 0.416 | 82.65% |
| BG | -0.158 | 0.069 | -0.364 | 0.067 | 90.07% |
| WV | -0.174 | 0.078 | -0.358 | 0.099 | 85.93% |

FS is filtering speed, WCL is wort clearness, EY is extract yield, WCO is wort color, BG is beta glucan, WV is wort viscosity; ^*^ Correlation that significantly different from 0.
